# Effectiveness of pressure biofeedback / pbu (pressure biofeedback unit) in the process of learning of self-correction in patients with scoliosis: a pilot study

**DOI:** 10.1186/1748-7161-8-S1-P10

**Published:** 2013-06-03

**Authors:** D Pennella, F Maselli, G Giovannico, M Cannone, A Rhainò, A Ciuro

**Affiliations:** 1 “San Raffaele - Cittadella della Carità” Taranto, Italy; 2 Genova University, Genova,Italy; 3 Padova University, Padova, Italy; 4 Fondazione Centri di riab. Padre Pio Foggia, Italy; 5 Ist. Sant’Agostino Noicattaro, Italy

## Introduction

The self-correction or active correction on all levels, in the treatment of scoliosis, is now a key tool with, or without, brace treatment [1]. The primary difficulty that the patient found is to understand which muscles to activate, and how to do it to achieve significant changes in the spine, since each patient adopts a personal strategy, hardly ever fair and efficient.

## Aim

The aim of this study is to verify the usefulness of the BPU to facilitate the learning processes of the Self-Correction, in patients with adolescent idiopathic scoliosis, in free or brace treatment. Through a BPU, each patient can be facilitated, by learning a right activation of the deep muscles of the spine [2], the clinician can also objectify the course of treatment.

## Methods

We enrolled 10 patients (5 for the experimental group, and 5 for control group) with adolescent idiopathic scoliosis, treated for up to 4 individual sessions of 40 minutes, according to a Self-correction of the scoliotic curve learning approach. Inclusion criteria:

- Adolescent Idiopathic Scoliosis

- Cobb degrees range between 15 ° and 30 °

- Patients with, or without, brace treatment

The control group, mean age 12.4 years, Risser 2.6 and degrees Cobb 14.8 °, was driven to the learning of Self-correction in the traditional way (verbal approach and passive movement) while with the experimental group, mean age 13.2 years, Risser 3 and degrees Cobb 15.2, we introduced using of the BPU to obtain the vertebral derotation [3]. Patients were subjected to analysis of posture with Formetric in 1 ^ seated (at rest) and 2^, 3^ and 4 ^ session (position of Self-correction), to evaluate the timing, and the ability to learn.

## Results

The experimental group used an average of 96 minutes (2.4 sessions) to learn Self-correction, unlike the control group, which required 120 minutes (3 sessions).

## Conclusions

The results of this pilot study define the usefulness of further research in this field, through an RCT of appropriate size.
